# 24(*S*),25-Epoxycholesterol and *cholesterol 24S-hydroxylase* (*CYP46A1*) overexpression promote midbrain dopaminergic neurogenesis *in vivo*

**DOI:** 10.1074/jbc.RA118.005639

**Published:** 2019-01-17

**Authors:** Spyridon Theofilopoulos, Willy Antoni Abreu de Oliveira, Shanzheng Yang, Eylan Yutuc, Ahmed Saeed, Jonas Abdel-Khalik, Abbe Ullgren, Angel Cedazo-Minguez, Ingemar Björkhem, Yuqin Wang, William J. Griffiths, Ernest Arenas

**Affiliations:** From the ‡ Laboratory of Molecular Neurobiology, Department of Medical Biochemistry and Biophysics, Karolinska Institutet, Stockholm 17177, Sweden,; the § Regenerative Neurobiology Laboratory, Swansea University Medical School, Institute of Life Science 1, Singleton Park, Swansea SA2 8PP, United Kingdom,; the ¶ Institute of Life Science, Swansea University Medical School, ILS1 Building, Singleton Park, Swansea SA2 8PP, United Kingdom,; the ‖ Division of Clinical Chemistry, Department of Laboratory Medicine, Karolinska Institutet and Karolinska University Hospital Huddinge, Stockholm 14157, Sweden, and; the ** Center for Alzheimer Research, Department of Neurobiology Care Sciences and Society, Division of Neurogeriatrics, Karolinska Institutet, Stockholm 14157, Sweden

**Keywords:** development, lipid metabolism, MS, neurodegenerative disease, neurogenesis, CYP46A1, dopamine neuron, liver X receptor, midbrain, oxysterol

## Abstract

The liver X receptors Lxrα/NR1H3 and Lxrβ/NR1H2 are ligand-dependent nuclear receptors critical for midbrain dopaminergic (mDA) neuron development. We found previously that 24(*S*),25-epoxycholesterol (24,25-EC), the most potent and abundant Lxr ligand in the developing mouse midbrain, promotes mDA neurogenesis *in vitro*. In this study, we demonstrate that 24,25-EC promotes mDA neurogenesis in an Lxr-dependent manner in the developing mouse midbrain *in vivo* and also prevents toxicity induced by the Lxr inhibitor geranylgeranyl pyrophosphate. Furthermore, using MS, we show that overexpression of human *cholesterol 24S-hydroxylase* (*CYP46A1*) increases the levels of both 24(*S*)-hydroxycholesterol (24-HC) and 24,25-EC in the developing midbrain, resulting in a specific increase in mDA neurogenesis *in vitro* and *in vivo*, but has no effect on oculomotor or red nucleus neurogenesis. 24-HC, unlike 24,25-EC, did not affect *in vitro* neurogenesis, indicating that the neurogenic effect of 24,25-EC on mDA neurons is specific. Combined, our results indicate that increased levels of 24,25-EC *in vivo*, by intracerebroventricular delivery in WT mice or by overexpression of its biosynthetic enzyme CYP46A1, specifically promote mDA neurogenesis. We propose that increasing the levels of 24,25-EC *in vivo* may be a useful strategy to combat the loss of mDA neurons in Parkinson's disease.

## Introduction

The vertebrate central nervous system is composed of an extensive variety of neurons that are generated following tightly regulated developmental programs. Characterization of the function and specificity of molecules selectively controlling distinct neuronal populations is thus essential to enhance our understanding of how such complexity is achieved in the developing brain, how it is maintained in the adult brain, and how it can be used for therapeutic purposes. Specific nuclear hormone receptors and their ligands have been identified as crucial factors in these processes (1–3). We have shown previously that liver X receptors (Lxrα^3^ and Lxrβ, encoded by *NR1H3* and *NR1H2*, respectively) and their endogenous brain ligands (oxidized derivatives of cholesterol and related molecules) regulate the development of midbrain dopamine (mDA) neurons (4–6), red nucleus neurons (5), as well as oculomotor neurons (7). Moreover, enzymes involved in the biosynthesis of cholesterol, oxysterols, and 24(*S*),25-epoxycholesterol (24,25-EC), such as 2,3-oxidosqualene-lanosterol cyclase, cytochrome P450 family 11 subfamily A member 1 (CYP11A1), and CYP46A1 (also known as cholesterol 24S-hydroxylase), are expressed in the developing mouse ventral midbrain (VM) during VM neurogenesis (4, 8, 9).^4^ The enzyme CYP46A1 oxidizes cholesterol to 24(*S*)-hydroxycholesterol (24-HC), the most abundant oxysterol in the adult brain, present at 20–40 ng/mg in the mouse and human (11). It has been shown, *in vitro* in human embryonic kidney 293 cells transfected with *CYP46A1*, that 24-HC can be further oxidized to 24,25-dihydroxycholesterol (24,25-diHC) and to 24,27-diHC (systematic name 24,26-diHC (12)) by CYP46A1 (13, 14). It has been suggested by these studies that 24,25-diHC could then be converted to 24,25-EC, but definite evidence for such a mechanism is lacking. Interestingly, it has been shown *in vitro* that CYP46A1 can also oxidize desmosterol to 24,25-EC and to 27-hydroxydesmosterol (systematic name 26-hydroxydesmosterol) (15), thereby providing a distinct 24,25-EC biosynthetic pathway via desmosterol in the brain. In agreement with this study, there is a reduction in both 24-HC and 24,25-EC levels in the *Cyp46a1*-knockout mouse adult brain compared with the WT brain (16). An alternative route to 24,25-EC formation is via a shunt of the mevalonate pathway, specifically the Bloch arm of the pathway, in which an extra oxygen atom is introduced by squalene epoxidase into 3S-squalene-2,3-epoxide to give squalene-2,3(S);22(S),23-diepoxide prior to cyclization by 2,3-oxidosqualene-lanosterol cyclase (17). This pathway is also expected to be impaired in the *Cyp46a1*-knockout mouse, as the necessary enzymes are down-regulated as a consequence of reduced cholesterol biosynthesis (18).

The functions of 24-HC and 24,25-EC in the central nervous system are diverse. 24-HC plays a role as a cholesterol transport molecule, crossing the blood–brain barrier and thus facilitating transport of cholesterol to the liver for further metabolism (8, 11, 19). 24-HC is also a ligand for Lxrα and Lxrβ in the brain (5) and binds to the endoplasmic reticulum–resident protein INSIG (insulin-induced gene) (20), modulating processing of SREBP-2 (sterol response element–binding protein-2) to its active form as the master transcription regulator for cholesterol biosynthesis. On the other hand, 24,25-EC is the most abundant Lxr ligand in the developing but not the adult brain (5, 21). Within the embryonic VM, 24,25-EC is present at a much higher concentration than 24-HC (Ref. 5 and this study). Moreover, we found previously that 24,25-EC is the most potent endogenous Lxr ligand to promote mDA neurogenesis in mouse progenitor VM cultures, embryonic stem cells *in vitro*, and zebrafish *in vivo* (5). However, the function of 24,25-EC in the developing mouse brain *in vivo* remains to be determined. In this study, we address this question by examining the midbrain of mouse embryos either injected intracerebroventricularly with 24,25-EC *in utero* or transgenic mice expressing *CYP46A1* under the control of a hybrid β-actin promoter (22). We show that increases in 24,25-EC in the developing VM, by either of these two strategies, result in increased number of mDA neurons *in vivo*. Thus, our results identify a new function of CYP46A1 and 24,25-EC in the mammalian brain *in vivo*.

## Results

### CYP46A1-overexpressing mice exhibit elevated levels of 24-HC and 24,25-EC

**Figure 1.**
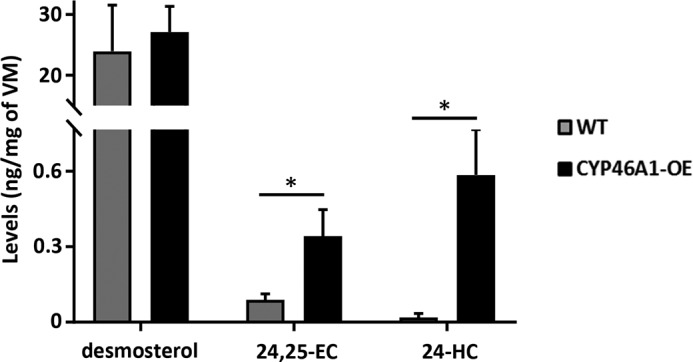
**CYP46A1-overexpressing mice exhibit elevated levels of 24,25-EC and 24-HC.** LC-MS(MS^n^) analysis demonstrated a significant increase in 24,25-EC and 24-HC but not desmosterol concentrations in the developing VM of *CYP46A1*-overexpressing mice compared with WT mice. Data are means ± S.E. (*n* = 4–6); *, *p* < 0.05 by Mann–Whitney test compared with the WT group.

To examine the role of CYP46A1 in the developing brain, we examined the VM of transgenic mice overexpressing this enzyme. We first analyzed the levels of several sterols, oxysterols, and related compounds in WT and *CYP46A1*-overexpressing mice. We found a 29.2-fold increase in 24-HC levels and a 3.9-fold increase in 24,25-EC levels in the developing VM of *CYP46A1*-overexpressing mice compared with WT mice at E11.5 (Fig. 1 and Table S1). We also found a 1.98-fold increase in cholesterol levels in the developing VM of *CYP46A1*-overexpressing mice compared with WT mice (Table S1). However, the level of desmosterol (266-fold higher than that of 24,25-EC in WT mice) did not change in *CYP46A1*-overexpressing mice. Furthermore, we did not find any alteration in 22(*R*)-HC, 25-HC, 27-HC (systematic name (25*R*)26-HC), 7α-HC, or 7α,24-dihydroxycholesterol in *CYP46A1*-overexpressing mice (Table S1), indicating that the increases in 24-HC and 24,25-EC levels are very specific.

To determine whether these changes were stable over time, we analyzed the levels of these compounds in the adult brain of *CYP46A1-*overexpressing mice. We found that, although the levels of cholesterol were not significantly different from WT mice, the levels of 24-HC and 24,25-EC increased by 22% and 25%, respectively, in *CYP46A1*-overexpressing mice (Table S2). Thus, our results portend CYP46A1 as a highly relevant enzyme in the biosynthesis of 24,25-EC in the developing and adult mouse brain.

Interestingly, our analysis of the developing mouse VM by single-cell RNA-Seq (9) indicates that *Cyp46a1* is expressed at higher levels in two cell types lining the ventricle, ependymal and radial glia–like3 cells (Fig. S1), suggesting that these cell types may be the endogenous source of 24-HC and 24,25-EC in the developing VM.

### Increased dopamine neuron number in midbrain cultures from CYP46A1-overexpressing mice

**Figure 2.**
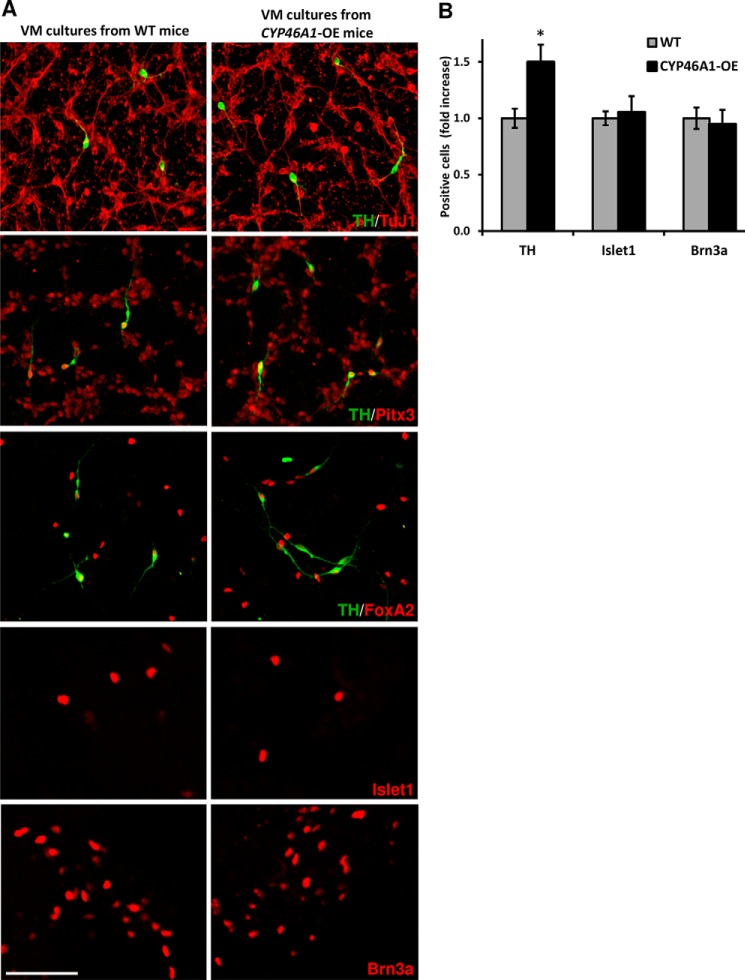
**Increased dopamine neuron numbers in midbrain cultures from *CYP46A1*-overexpressing mice.** A, representative images of TH^+^ and TuJ1^+^ neurons as well as Foxa2^+^, Pitx3^+^, Islet1^+^, and Brn3a^+^ neuron nuclei in VM cultures from WT and *CYP46A1*-overexpressing mice. *Scale bar* = 50 μm. *B*, quantification of TH^+^, Islet1^+^, and Brn3a^+^ neurons in VM cultures from WT and *CYP46A1*-overexpressing mice. Data are means ± S.E. (*n* = 3); *, *p* < 0.05 by Student's *t* test compared with the WT group.

We next studied the impact of *CYP46A1* overexpression on distinct neuronal populations in the developing VM. Notably, mouse VM progenitor cultures from *CYP46A1*-overexpressing mice exhibited a significant 49.8% increase in the number of mDA neurons compared with cultures from WT mice (Fig. 2, *A* and *B*). These neurons co-expressed the rate-limiting enzyme in the synthesis of dopamine tyrosine hydroxylase (TH), β III tubulin (TuJ1, a pan-neuronal marker), Forkhead box transcription factor (Foxa2, required for midbrain development regulation) (23), and pituitary homeobox 3 (Pitx3, a transcription factor required for the survival and maintenance of mDA neurons) (24) (Fig. 2*A*), thereby showing that they were true mDA neurons. Because mouse *Cyp46a1* was also expressed in other cell types of the developing VM (Fig. S1), we examined adjacent neuronal populations. No significant change in the number of Islet1^+^ oculomotor neurons or Brn3a^+^ red nucleus neurons was detected (Fig. 2*B*), thereby demonstrating that the effect of *CYP46A1* overexpression is specific to mDA neurons. We next examined whether 24-HC and 24,25-EC increase the number of mDA neurons when added to WT VM progenitor cultures. Although 24,25-EC enhances mDA neurogenesis (Ref. 5 and Fig. S2), we found that 24-HC had no significant effect on the number of TH^+^ mDA neurons (Fig. S3). Interestingly, the effect of 24,25-EC on TH^+^ mDA neurons was abolished in VM progenitor cultures from *Lxr*αβ double knockout mice (Fig. S2), thereby showing that LXR receptors are required for the increase in mDA neuron numbers by 24,25-EC. Combined, our results indicate that elevated levels of 24,25-EC lead to increased numbers of mDA neurons in *CYP46A1*-overexpressing mice.

### CYP46A1 overexpression increases the number of mDA neurons in the developing brain in vivo

**Figure 3.**
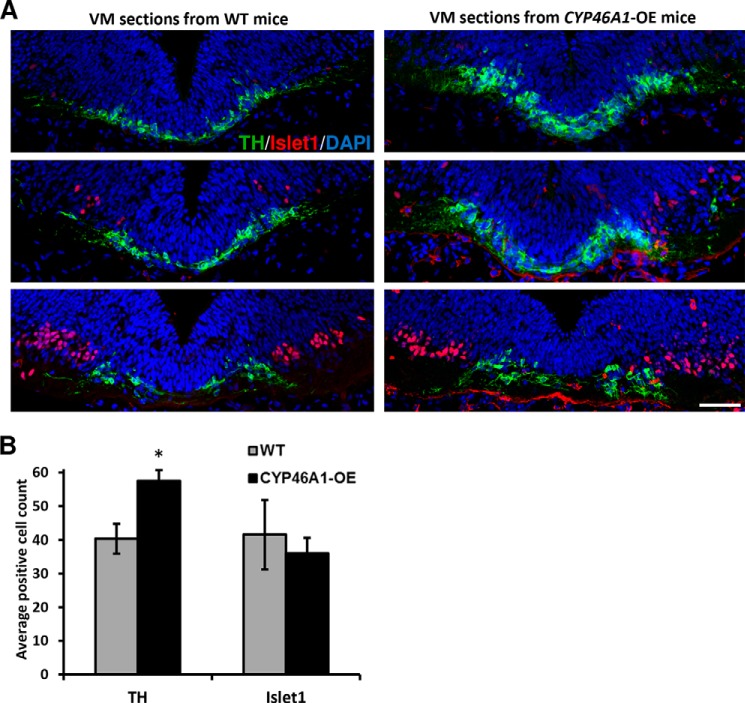
**Increased dopamine neuron numbers in the VM of *CYP46A1*-overexpressing mice.** *A*, representative images of anterior-to-posterior coronal VM sections from E11.5 WT and *CYP46A1*-overexpressing mice showing TH^+^ neurons, Islet1^+^ neuron nuclei, and 4′,6-diamidino-2-phenylindole–stained nuclei. *Scale bar* = 50 μm. *B*, quantification of TH^+^ and Islet1^+^ neurons in VM sections from WT and *CYP46A1*-overexpressing mice. Data are means ± S.E. (*n* = 5–15); *, *p* < 0.05 by Mann–Whitney test compared with the WT group.

We also investigated whether *CYP46A1*-overexpression impacts VM development *in vivo*. We thus examined the number of mDA and oculomotor neurons in coronal sections through the VM of *CYP46A1*-overexpressing and WT mice at E11.5. We observed that the number of TH^+^ mDA neurons significantly increased by 42.6% in *CYP46A1*-overexpressing compared with WT mice (Fig. 3, *A* and *B*). However, the number of Islet1^+^ oculomotor neurons did not change, arguing for a specific effect of *CYP46A1* overexpression on mDA neurons *in vivo*. Combined, our results indicate that elevated levels of 24,25-EC in *CYP46A1*-overexpressing mice lead to increased numbers of mDA neurons.

### 24,25-EC promotes mouse midbrain dopaminergic neurogenesis in vivo and prevents toxicity by GGPP

**Figure 4.**
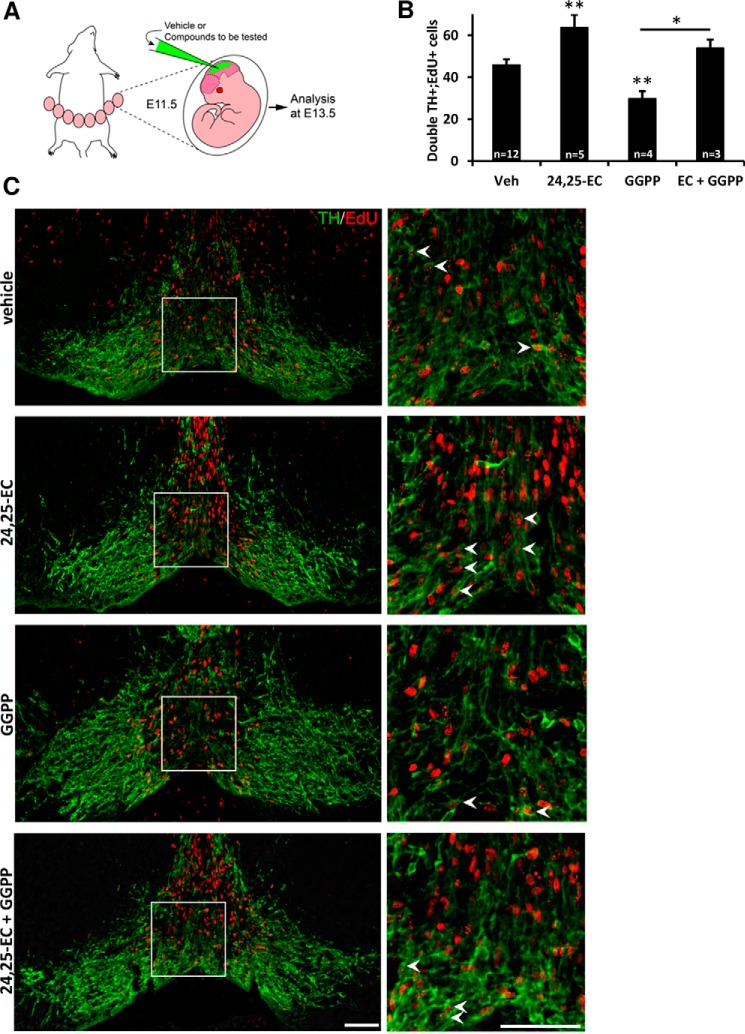
**24,25-EC promotes neurogenesis of mouse midbrain dopamine neurons *in vivo* and prevents toxicity by GGPP.** *A*, vehicle or 24,25-EC and/or GGPP was injected into the mesencephalic ventricle of E11.5 mouse embryos *in utero*, and embryos were collected at E13.5. Dopamine neurogenesis was examined by EdU intraperitoneal injection and by assessing the acquisition of TH expression. *B*, quantification of double EdU^+^;TH^+^ cell numbers (mean ± S.E.) for the indicated conditions: vehicle, 24,25-EC-, GGPP-, and 24,25-EC + GGPP–injected embryos. Data are means ± S.E. (*n* = 3–12); *, *p* < 0.05; **, *p* < 0.01 by Mann–Whitney test compared with the vehicle group or as indicated. *C*, microphotographs of midbrain coronal sections showing TH^+^ dopamine neurons (*green*) and EdU^+^ cells (*red*) (*left panels*) and higher-magnification pictures of the *boxed region* (*right panels*) for the indicated conditions. *Arrowheads* indicate double EdU^+^;TH^+^ cells. *Scale bars* = 50 μm.

Finally, to directly examine the function of 24,25-EC in the developing mouse midbrain *in vivo*, we performed 24,25-EC injections into the cerebrospinal fluid, at the level of the aqueduct, in E11.5 WT mouse embryos *in utero* and analyzed brain sections at the midbrain level at E13.5 (Fig. 4*A*). Neurogenesis was examined by performing a pulse of EdU intraperitoneally at E11.5 to label proliferative progenitors and assess their capacity to undergo neurogenesis and give rise to mDA neurons that can be identified by the expression of *tyrosine hydroxylase* (*Th*). Upon injection of 24,25-EC, we found that the number of double EdU^+^;TH^+^ cells increased by 39% (Fig. 4, *B* and *C*), thereby demonstrating that 24,25-EC promotes mDA neurogenesis *in vivo*. In contrast, injection of the Lxr inhibitor geranylgeranyl pyrophosphate (GGPP), reduced the number of double EdU^+^;TH^+^ cells, indicating that LXR activity is required for mDA neuron development. Notably, the effect of GGPP was blocked by co-injection of 24,25-EC, indicating that 24,25-EC is not only required and sufficient to promote mDA neurogenesis *in vivo* but can also prevent the toxic effect of GGPP. Thus, our results demonstrate that elevated levels of 24,25-EC promote mDA neurogenesis *in vivo*.

## Discussion

In this study, we show that overexpression of *CYP46A1* in transgenic mice increases the levels of 24-HC and 24,25-EC in the VM but does not alter desmosterol or other oxysterol levels, which remain at a similar level as in WT mice. Our results, together with previous findings showing a reduction in both 24-HC and 24,25-EC levels in *Cyp46a1*-knockout mice (16), lend support to the hypothesis that CYP46A1 is highly relevant in the biosynthesis of 24,25-EC. This could be achieved either by increased biosynthesis of 24,25-EC from desmosterol by CYP46A1 (as suggested in Ref. 15) or by increased biosynthesis of 24,25-EC from cholesterol by CYP46A1 via 24-HC and 24,25-diHC (as suggested in Refs. 13, 14). In either case, our results demonstrate the importance of CYP46A1 in 24,25-EC biosynthesis in the developing mammalian VM. We also found that 24-HC does not affect the number of mDA neurons, whereas 24,25-EC strongly promotes mDA neurogenesis, a finding consistent with our previous results showing Lxr ligand–specific activities in the developing mouse VM (5, 7). These results also indicate that the observed increase in mDA neurogenesis *in vitro* and *in vivo* in *CYP46A1*-overexpressing mice is indeed associated with the increase in 24,25-EC in these mice. Notably, the effect of *CYP46A1* was specific to mDA neurons, as neighboring cell populations in the developing basal plate of the VM, such as oculomotor neurons or red nucleus neurons, were not affected in *CYP46A1*-overexpressing mice. These results show that increased levels of Lxr ligands do not alter their cell type specificity, which is conferred by LXRs, as we described previously (5, 7). Mechanistically, we found that intracerebroventricular injection of the LXR agonist 24,25-EC or the LXR antagonist GGPP was capable of, respectively, promoting or inhibiting mDA neurogenesis *in vivo*. These effects were specific because 24,25-EC had no effect on red nucleus, serotonin^+^ neurons, oculomotor neurons, or GABA^+^ neurons *in vitro* and *in vivo* (Ref. 5 and this work). In sum, our results demonstrate a clear role of LXR receptors and 24,25-EC in mDA neurogenesis *in vivo*.

Several studies have associated a reduced level of CYP46A1 with neurodegeneration and neuronal dysfunction as well as restoration of normal CYP46A1 levels with functional recovery and neuroprotection. For instance, knockdown of *Cyp46a1* in mice results in deficits in spatial, associative, and motor learning and in hippocampal long-term potentiation (25). In addition, reduced *Cyp46a1* levels result in cognitive deficits, elevated production of β-amyloid peptides, and abnormal phosphorylation of tau (26) as well as in progressive loss of hippocampal neurons and an Alzheimer's disease–like phenotype (27). Conversely, increased expression of *CYP46A1* improves spatial memory retention in aged female mice (28) and reduces cognitive decline and amyloid burden in several mouse models of Alzheimer's disease (29–31). Furthermore, similar results have been obtained by enhancing CYP46A1 activity with the reverse transcriptase inhibitor efavirenz (32), arguing for the feasibility of using a pharmacological treatment to reduce neurodegeneration.

With regard to neurodegeneration in the basal ganglia, it has been reported that the level of CYP46A1 is decreased in the putamen of patients with Huntington's disease (33). Notably, CYP46A1 knockdown in the mouse striatum induced spontaneous striatal neurodegeneration associated with abnormal balance and motor coordination. Conversely, increased levels of CYP46A1 in the R6/2 Huntington's disease mouse model decreased striatal neuron atrophy, protein aggregates, and motor deficits.

Much less is known about the role of CYP46A1 in Parkinson's disease. For instance, it remains to be determined whether the level and functionality of CYP46A1 are conserved or altered in Parkinson's disease models or patients. The results in this study provide first evidence that 24,25-EC can rescue a defect in mDA neurogenesis induced by GGPP *in vivo*, suggesting a potential application of CYP46A1 and 24,25-EC in regenerating mDA neurons *in vivo*. Previous studies have also shown that a synthetic LXR ligand can prevent the degeneration of mDA neurons in an animal model of Parkinson's disease (34). Thus combined, our results and data in the literature suggest that 24,25-EC or pharmacological tools capable of activating LXR receptors or enhancing the function and/or levels of CYP46A1 could be used to enhance mDA neurogenesis, limit neurodegeneration, and advance cell replacement strategies for the treatment of Parkinson's disease.

## Experimental procedures

### Extraction of sterols

Sterols were extracted from mouse adult brain and mouse embryonic VM into ethanol and fractionated by reverse-phase solid phase extraction (SPE) to give an oxysterol-rich fraction devoid of cholesterol (5, 7, 16, 36).

### Charge-tagging of sterols

The sterols were charge-tagged with GP-hydrazine as described previously (5, 7, 16, 36). This greatly enhances their response when analyzed by LC-electrospray ionization-MS (LC-ESI-MS) and MS with multistage fragmentation (MS^n^).

### Reagents

HPLC-grade water and solvents were from Fisher Scientific or Sigma-Aldrich. Authentic sterols and oxysterols were from Avanti Polar Lipids. Girard P (GP) reagent (1-[carboxymethyl]pyridinium chloride hydrazide, [^2^H_0_]GP) was from TCI Europe or synthesized in-house ([^2^H_5_]GP) as in earlier studies (37), and cholesterol oxidase from *Streptomyces* sp. was from Sigma-Aldrich. Certified Sep-Pak C_18_ 200-mg (SPE1) and OASIS HLB 60-mg (SPE2) columns were from Waters.

### LC-ESI-MS^n^ on the Orbitrap ELITE

LC-ESI-MS and LC-ESI-MS^n^ were performed using an Ultimate 3000 HPLC system (Dionex, now Thermo Fisher Scientific) linked to the ESI source of an Orbitrap ELITE (Thermo Fisher Scientific) mass spectrometer as described previously (5, 7, 16, 35, 36).

### WT mice

Mice were housed, bred, and treated according to the guidelines of the European Communities Council (directive 86/609/EEC) and the Society for Neuroscience. Ethics approval for mouse experimentation was granted by Stockholm Norra Djurförsöksetisks Nämnd N154/06, N145/09, N370/09, N273/11, and N486/12.

### Mice overexpressing human CYP46A1

Human *CYP46A1*-overexpressing transgenic mice were generated as described before (22, 28). All animal experiments received full approval from the local Animal Experimentation Ethics Committee. Tissue sampling from these mice was performed under the aegis of the UK Scientific Procedures (Animals) Act, 1986.

### Primary midbrain cultures

Brains from E11.5 mice were obtained. The ventral midbrain region was dissected, mechanically dissociated, plated on poly-d-lysine (150,000 cells/cm^2^), and grown in serum-free N2 media consisting of a 1:1 mixture of F12 and Dulbecco's modified Eagle's medium with 10 ng/ml insulin, 100 μg/ml apo-transferrin, 100 μm putrescine, 20 nm progesterone, 30 nm selenium, 6 mg/ml glucose, and 1 mg/ml BSA. Cells were treated for 3 days *in vitro* with the compounds of interest, fixed with 4% PFA, and processed for staining using appropriate antibodies. Hoechst staining was performed by permeabilizing cells with a 0.3% Triton X-100/PBS solution for 5 min, followed by incubation with Hoechst 33258 (Sigma) for 10 min.

### Immunocytochemical analysis

Cells were fixed in 4% paraformaldehyde (PFA), washed in PBS, and blocked in 5% normal goat serum/PBS for 1 h at room temperature. Primary antibodies were diluted in PBS (pH 7.4), 0.3% Triton X-100, and 1% BSA, and incubations were carried out overnight at +4 °C or at room temperature for 2 h. The antibodies used were anti-TH (1:1000, Pel-Freeze), anti-Islet1 (1:100, Developmental Studies Hybridoma Bank), anti-Brn3a (1:250, Millipore), anti-TuJ1 (1:1000, Promega), anti-FoxA2 (1:400, Cell Signaling Technology), anti-Pitx3 (1:400, Invitrogen) and appropriate secondary antibodies (Jackson ImmunoResearch Laboratories or Alexa). Cells positive for the corresponding marker were counted directly at the microscope at a magnification of ×20. Cells were counted in every well, in eight consecutive fields (going from one side of the well to the other, passing through the center), in three different wells per experiment, and in three different experiments per condition. Positive cell counts were normalized to the total number of cells (counted utilizing Hoechst-stained nuclei) and presented as -fold increase over WT or vehicle. Random pictures of the wells were taken for every condition to document the result, and representative pictures were subsequently selected to represent the quantitative data. Photos were acquired with a Zeiss Axioplan microscope and a Hamamatsu camera (C4742-95) using the Openlab software.

### In utero intraventricular injections

Mouse *in utero* injections were performed as described previously (7, 38). Female WT *CD-1* mice (25–35 g, Charles River Breeding Laboratories) were used for these experiments. Ethics approval was granted by Stockholm Norra Djurförsöksetisks Nämnd N273/11 and N486/12. For embryo analyses, WT *CD-1* mice were mated overnight, and noon of the day the plug was considered E0.5. E11.5 pregnant females were deeply anesthetized using isoflurane (IsoFlo®, Abbott Labs), and the uterine horns were accessed through an abdominal incision. 1 μl of 24,25-EC (5 mm), GGPP (5 mm), or vehicle solution (methanol/PBS, 50% v/v) was injected into the cerebral aqueduct. The uterine horns were replaced into the abdominal cavity, which was then closed with sutures. For EdU pulse-chase experiments, EdU (50 mg/kg of body weight) was injected by intraperitoneal injection 30 min after the injections to the embryo. Embryos were analyzed 48 h later, at E13.5. The concentration and volume of the compounds utilized in these experiments were chosen for the compounds to be in a physiological range because the cerebrospinal fluid volume in the E11.5 mouse embryo is ∼40 μl, the cerebrospinal fluid is replaced at a speed of 3.3 × 10^−4^ ml/min in mice (10), and mouse embryos were analyzed 48 h after injection.

### Mouse VM coronal sections and immunohistochemical analysis

Embryos were dissected out of the uterine horns in ice-cold PBS, fixed in 4% PFA for 4 h to overnight, cryoprotected in 15–30% sucrose, frozen in Tissue-Tek Optimum Cutting Temperature compound (Sakura Fine-Tek) on dry ice, and stored at −80 °C until use. 14-μm serial coronal sections through the E11.5 or E13.5 midbrain region were cut on a cryostat and placed serially on 10 slides. Slides 1 and 6 were subjected to immunohistochemistry. Sections were preincubated for 1 h in blocking solution, followed by incubation at 4 °C overnight with the following primary antibodies: sheep anti-TH (1:500, Novus Biologicals), rabbit anti-TH (1:750, Pel-Freeze), and mouse anti-Islet-1 (1:100, Developmental Studies Hybridoma Bank). After washing, slides were incubated for 1 h at room temperature with the appropriate fluorophore-conjugated (Cy2, Cy3, and Cy5, 1:300, Jackson ImmunoResearch Laboratories; Alexa 488, 555, and 647, 1:1000, Invitrogen) secondary antibodies. The EdU click reaction was performed according to the instructions of the manufacturer (Life Technologies). Confocal pictures were taken on a Zeiss LSM700 microscope. TH^+^ cells, Islet1^+^ cells, and double EdU^+^;TH^+^ cells were counted on three sections covering the rostral to caudal midbrain for each embryo.

### Statistical analysis

Statistical analyses (Mann–Whitney test and Student's *t* test) were performed using Prism 4 (GraphPad Software, La Jolla, CA). A *p* value less than 0.05 was considered significant; *, *p* < 0.05; **, *p* < 0.01. Data represent mean ± S.E.

### Animal studies approval statement

Ethics approval for WT and *CYP46A1*-overexpressing transgenic mouse experimentation was granted by the local Animal Experimentation Ethics Committee (Stockholm Djurförsöksetisks Nämnd N154/06, N145/09, N370/09, N273/11, and N486/12).

## Author contributions

S. T., I. B., Y. W., W. J. G., and E. A. conceptualization; S. T., W. A. A. d. O., S. Y., E. Y., A. S., J. A.-K., and A. U. data curation; S. T., W. A. A. d. O., S. Y., E. Y., A. S., J. A.-K., I. B., Y. W., W. J. G., and E. A. formal analysis; S. T., I. B., Y. W., W. J. G., and E. A. supervision; S. T., I. B., Y. W., W. J. G., and E. A. funding acquisition; S. T., W. A. A. d. O., S. Y., E. Y., A. S., J. A.-K., A. U., A. C.-M., I. B., Y. W., W. J. G., and E. A. investigation; S. T. and W. A. A. d. O. visualization; S. T. methodology; S. T. writing-original draft; S. T., I. B., Y. W., W. J. G., and E. A. project administration; S. T., I. B., Y. W., W. J. G., and E. A. writing-review and editing; A. C.-M., I. B., Y. W., W. J. G., and E. A. resources.

## Supplementary Material

Supporting Information

## Footnotes

4 The array data have been deposited in the NCBI Gene Expression Omnibus and are accessible through GEO accession number GSE76381 (https://github.com/linnarsson-lab/ipynb-lamanno2016).

## Footnotes

3 The abbreviations used are:

Lxr: liver X receptor
mDA: midbrain dopamine
24,25-EC: 24(*S*),25-epoxycholesterol
VM: ventral midbrain
24-HC: 24(*S*)-hydroxycholesterol
24,25-diHC: 24,25-dihydroxycholesterol
E11.5: embryonic day 11.5
TH: tyrosine hydroxylase
GGPP: geranylgeranyl pyrophosphate
SPE: solid phase extraction
GP: Girard P
ESI: electrospray ionization
MS^n^: MS with multistage fragmentation
PFA: paraformaldehyde
EdU: 5-ethynyl-2'-deoxyuridine.
