# Supplementary material for: 24(S),25-Epoxycholesterol and cholesterol 24S-hydroxylase (CYP46A1) overexpression promote midbrain dopaminergic neurogenesis in vivo
Source: J Biol Chem. 2019 Jan 17;294(11):4169–76. doi: 10.1074/jbc.RA118.005639 (PMC6422085; doi:10.1074/jbc.RA118.005639)
Supplement: Supporting Information [file supp_294_11_4169__index.html]

24(S),25-Epoxycholesterol and cholesterol 24S-hydroxylase (CYP46A1) overexpression promote midbrain dopaminergic neurogenesis in vivo — 24,25-Epoxycholesterol in the mouse brain — Supporting Information 

# 24(*S*),25-Epoxycholesterol and *cholesterol 24S-hydroxylase* (*CYP46A1*) overexpression promote midbrain dopaminergic neurogenesis *in vivo*

## Supporting Information

- Supporting Information - Supporting Information to the manuscript.
