# Supplementary material for: 24(S),25-Epoxycholesterol and cholesterol 24S-hydroxylase (CYP46A1) overexpression promote midbrain dopaminergic neurogenesis in vivo
Source: J Biol Chem. 2019 Jan 17;294(11):4169–76. doi: 10.1074/jbc.RA118.005639 (PMC6422085; doi:10.1074/jbc.RA118.005639)
Supplement: Supporting Information [file supp_RA118.005639_140482_2_supp_269055_plfqnx.pdf]

24(*S*),25-Epoxycholesterol and *cholesterol 24S-hydroxylase* (*CYP46A1*) overexpression promote midbrain dopaminergic neurogenesis *in vivo*

Spyridon Theofilopoulos[1,2,*], Willy Antoni Abreu de Oliveira[1], Shanzheng Yang[1], Eylan Yutuc[3], Ahmed Saeed[4], Jonas Abdel-Khalik[3], Abbe Ullgren[1,5], Angel Cedazo-Minguez[5], Ingemar Björkhem[4], Yuqin Wang[3], William J. Griffiths[3], and Ernest Arenas[1,*]

From the [1]Laboratory of Molecular Neurobiology, Department of Medical Biochemistry and Biophysics, Karolinska Institutet, Stockholm 17177, Sweden; [2]Regenerative Neurobiology laboratory, Swansea University Medical School, Institute of Life Science 1, Singleton Park, Swansea SA2 8PP, United Kingdom; [3]Institute of Life Science, Swansea University Medical School, ILS1 Building, Singleton Park, Swansea SA2 8PP, United Kingdom; [4]Division of Clinical Chemistry, Department of Laboratory Medicine, Karolinska Institutet and Karolinska University Hospital Huddinge, Stockholm 14157, Sweden; [5]Center for Alzheimer Research, Department of Neurobiology Care Sciences and Society, Division of Neurogeriatrics, Karolinska Institutet, Stockholm 14157, Sweden.

Running title: *24,25-Epoxycholesterol in the mouse brain*

*To whom correspondence should be addressed:
Spyridon Theofilopoulos, Regenerative Neurobiology laboratory, Institute of Life Science 1, Swansea University Medical School, Swansea SA2 8PP, UK. Tel: +44 1792513603; E-mail: s.theofilopoulos@swansea.ac.uk.
Or to:
Ernest Arenas, Laboratory of Molecular Neurobiology, Department of Medical Biochemistry and Biophysics, Karolinska Institutet, Stockholm, 17177, Sweden. Tel: +46 852487663; E-mail: ernest.arenas@ki.se.

<u>Supporting Information table of contents:</u>

Table S1. Oxysterols and related compounds identified by LC-MS^n in ventral midbrain of E11.5 wild-type and *CYP46A1*-overexpressing mice.

Table S2. 24(*S*)-hydroxycholesterol, 24(*S*),25-epoxycholesterol and cholesterol levels, identified and quantified by LC-MS, in the adult brain of wild-type and *CYP46A1*-overexpressing mice.

Figure S1. Expression of *Cyp46a1* in the wild-type mouse ventral midbrain as assessed by single cell RNA-sequencing

Figure S2. LXR receptors are required for the increase of mDA neuron numbers by 24,25-EC.

Figure S3. 24-HC does not affect the number of midbrain dopamine neurons in culture.

Table S1. Oxysterols and related compounds identified by LC-MS^n in ventral midbrain (VM) of E11.5 wild-type (n=4) and *CYP46A1*-overexpressing (n=6) mice.

		**VM[a]**						
**Identified Structure after Treatment with Cholesterol Oxidase**	**$[M]^+$ of GP[a] *m/z***	**WT (ng/mg of VM)**	***CYP46A1*-OE (ng/mg of VM)**	**Rt (min)**	***p*-value**	**Inferred Structure prior to Treatment with Cholesterol Oxidase**	**Inferred Compound Trivial Name**	**Comments/ Parameters for Identification**
C-4-en-24S,25-epoxide-3-one	532.3898	0.004 ± 0.002	0.016 ± 0.005	6.86/7.08	NS	C-5-en-3β-ol-24S,25-epoxide	24(*S*),25-epoxycholesterol	Appear as *syn* and *anti* conformers, Rt, MS, MS^n
C-4-en-3,24-dione[b]	532.3898	0.017 ± 0.004	0.063 ± 0.027	7.83/7.91	NS	C-5-en-3β-ol-24S,25-epoxide	24(*S*),25-epoxycholesterol	Rt, MS, MS^n
C-4-en-24,25-diol-3-one[c]	550.4003	0.037 ± 0.007	0.153 ± 0.038	4.14/4.78	0.009	C-5-en-3β-ol-24S,25-epoxide	24(*S*),25-epoxycholesterol	Appear as *syn* and *anti* conformers, Rt, MS, MS^n
C-4-en-24-ol,25-OMe-3-one[d]	564.4160	0.032 ± 0.009	0.123 ± 0.034	6.25/6.67	0.038	C-5-en-3β-ol-24S,25-epoxide	24(*S*),25-epoxycholesterol	Appear as *syn* and *anti* conformers, Rt, MS, MS^n
		**0.090 ± 0.023**	**0.355 ± 0.099**		**0.012**	**C-5-en-3β-ol-24S,25-epoxide**	**24(*S*),25-epoxycholesterol**	**Total 24(*S*),25-Epoxycholesterol**
C-4-en-22R-ol-3-one	534.4054	0.001 ± 0.0006	0.001 ± 0.0003	6.22	NS	C-5-en-3β,22R-diol	22(*R*)-hydroxycholesterol	Rt, MS, MS^n
C-4-en-24S-ol-3-one	534.4054	0.020 ±0.014	0.585 ± 0.180	7.18/7.58	0.011	C-5-en-3β,24S-diol	24(*S*)-hydroxycholesterol	Appear as *syn* and *anti* conformers, Rt, MS, MS^n
C-4-en-25-ol-3-one	534.4054	0.005 ± 0.001	0.006 ± 0.001	7.53	NS	C-5-en-3β,25-diol	25-hydroxycholesterol	Rt, MS, MS^n

C-4-en-26-ol-3-one	534.4054	0.004 ±0.001	0.005 ± 0.001	8.03/8.12	NS	C-5-en-3β,(25R)26-diol[e]	27-hydroxycholesterol[e]	Appear as *syn* and *anti* conformers, Rt, MS, MS^n
C-4-en-7α-ol-3-one	534.4054	0.011 ± 0.002	0.010 ± 0.002	10.20	NS	C-5-en-3β,7α-diol	7α-hydroxycholesterol[f]	Rt, MS, MS^n
C-4-en-7α,24-diol-3-one	550.4003	0.014 ± 0.002	0.020 ± 0.002	5.71/6.69	NS	C-5-en-3β,7α,24-triol	7α,24-di-hydroxycholesterol	Appear as *syn* and *anti* conformers, Rt, MS, MS^n
C-4,24-dien-3-one	516.3948	23.98 ± 7.541	27.11 ± 4.192	11.06	NS	C-5,24-dien-3β-ol	desmosterol	Rt, MS, MS^n
C-4-en-3-one	518.4105	338.1 ± 60.61	669.3 ± 76.07	12.15	0.019	C-5-en-3β-ol	cholesterol	Rt, MS, MS^n

Footnotes to the table:
Systematic nomenclature adopted according to Lipid Maps http://www.lipidmaps.org/ .
C = cholestane, a number preceding “en” indicates the location of carbon-carbon double bond(s), a number(s) preceding “ol (diol, etc)” or “one” indicates the location of hydroxy and oxo groups, respectively.
Rt = retention time; No reference = no authentic standard available; NS = non-significant difference by Mann-Whitney test.
[a] Data for GP derivatives.
[b] Isomerisation product of C-4-en-24S,25-epoxide-3-one.
[c] Hydrolysis product of C-4-en-24S,25-epoxide-3-one.
[d] Alternatively C-4-en-25-ol,24-OMe-3-one, methanolysis product of C-4-en-24S,25-epoxide-3-one
[e] According to systematic nomenclature recommended by the Lipid Maps consortium, hydroxylation of the terminal carbon of cholesterol introducing 25R stereochemistry is at C-26 leading to C-5-en-3β,(25R)26-diol. However, the common name is 27-hydroxycholesterol.
[f] Formed enzymatically by CYP7A1 and/or by autoxidation.
[n] MS/MS or MS/MS/MS

Table S2. 24(*S*)-hydroxycholesterol, 24(*S*),25-epoxycholesterol and cholesterol levels, identified and quantified by LC-MS, in the adult brain of wild-type (n=4) and *CYP46A1*-overexpressing (n=3) mice.

	**WT (ng/µg cholesterol)**	***CYP46A1*-OE (ng/µg cholesterol)**	***CYP46A1*-OE / WT**	***p*-value**
24(*S*)-hydroxycholesterol	2.148 ± 0.057	2.614 ± 0.118	1.22 ± 0.06	0.002
24(*S*),25-epoxycholesterol	0.078 ± 0.008	0.097 ± 0.008	1.25 ± 0.16	0.042

	**WT (ng/µg of brain)**	***CYP46A1*-OE (ng/µg of brain)**	***CYP46A1*-OE / WT**	***p*-value**
cholesterol	10.823 ± 0.573	10.457 ± 0.999	0.97 ± 0.11	> 0.05

Figure S1

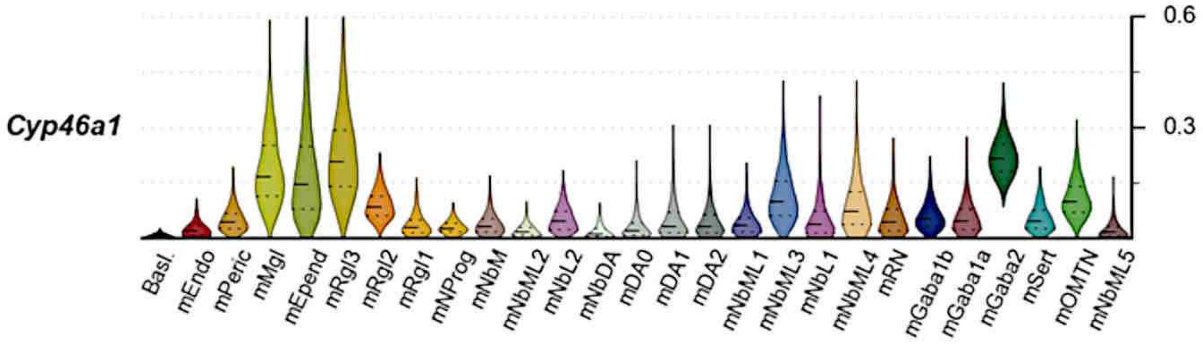

Figure S1. Expression of *Cyp46a1* in the wild-type mouse ventral midbrain as assessed by single cell RNA-sequencing. Two cell types lining the ventricle, ependymal (mEpend) and radial glia-like3 (mRgl3) cells, as well as microglia (mMgl) and a subtype of GABAergic neurons (mGABA2), express higher levels of *Cyp46a1* compared to all other cell types in the embryonic VM. For a list of all cell type abbreviations see (9).

Figure S2

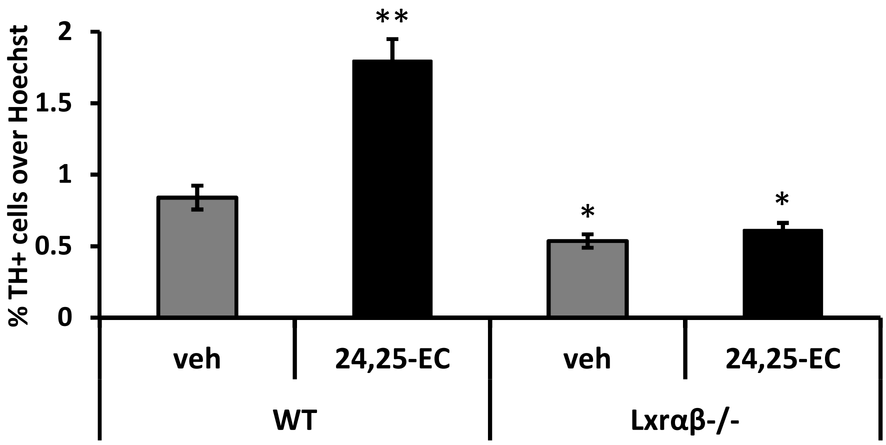

Figure S2. LXR receptors are required for the increase of mDA neuron numbers by 24,25-EC. The effect of 24,25-EC on TH^+ mDA neurons was abolished in VM progenitor cultures from *Lxrαβ* double knockout mice. Data are means ± SEM (n = 3), * $P < 0.05$, ** $P < 0.01$ by Student's *t*-test, compared to WT vehicle group.

Figure S3

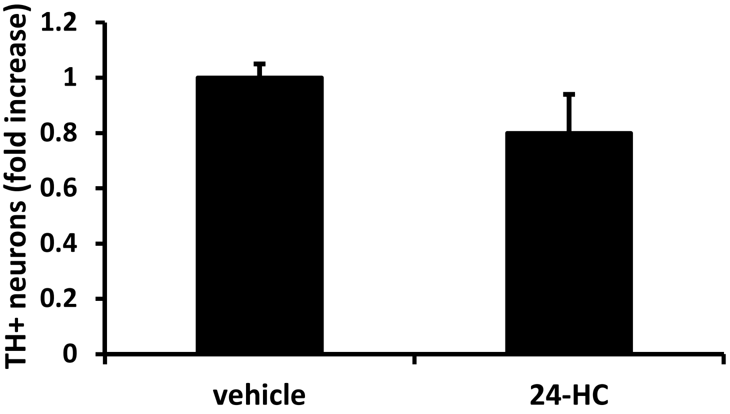

Figure S3. 24-HC does not affect the number of midbrain dopamine neurons in culture. Quantification of TH^+ neurons in wild-type VM cultures treated with vehicle or 24-HC. Data are means ± SEM (n = 6).
